# MicroRNA Regulation of Brain Tumour Initiating Cells in Central Nervous System Tumours

**DOI:** 10.1155/2015/141793

**Published:** 2015-05-03

**Authors:** Neha Garg, Thusyanth Vijayakumar, David Bakhshinyan, Chitra Venugopal, Sheila K. Singh

**Affiliations:** ^1^McMaster Stem Cell and Cancer Research Institute, McMaster University, Hamilton, ON, Canada L8S 4K1; ^2^Department of Surgery, Faculty of Health Sciences, McMaster University, 1200 Main Street West, Hamilton, ON, Canada L8N 3Z5; ^3^Department of Biochemistry and Biomedical Sciences, Faculty of Health Sciences, McMaster University, 1200 Main Street West, Hamilton, ON, Canada L8N 3Z5; ^4^Michael G. DeGroote School of Medicine, McMaster University, 1200 Main Street West, Hamilton, ON, Canada L8N 3Z5

## Abstract

CNS tumours occur in both pediatric and adult patients and many of these tumours are associated with poor clinical outcome. Due to a paradigm shift in thinking for the last several years, these tumours are now considered to originate from a small population of stem-like cells within the bulk tumour tissue. These cells, termed as brain tumour initiating cells (BTICs), are perceived to be regulated by microRNAs at the posttranscriptional/translational levels. Proliferation, stemness, differentiation, invasion, angiogenesis, metastasis, apoptosis, and cell cycle constitute some of the significant processes modulated by microRNAs in cancer initiation and progression. Characterization and functional studies on oncogenic or tumour suppressive microRNAs are made possible because of developments in sequencing and microarray techniques. In the current review, we bring recent knowledge of the role of microRNAs in BTIC formation and therapy. Special attention is paid to two highly aggressive and well-characterized brain tumours: gliomas and medulloblastoma. As microRNA seems to be altered in the pathogenesis of many human diseases, “microRNA therapy” may now have potential to improve outcomes for brain tumour patients. In this rapidly evolving field, further understanding of miRNA biology and its contribution towards cancer can be mined for new therapeutic tools.

## 1. Introduction

MicroRNAs are small (19–25 nucleotides) noncoding RNAs that bind within the 3′ untranslated region (UTR) of protein coding mRNAs [1] and regulate gene expression. This sequence-dependent posttranscriptional regulation of gene expression occurs either by repressing translation or degradation of target mRNAs [2]. Recently, a novel regulatory mechanism to regulate transcription or stimulate translation by binding to gene promoters or 3′- and 5′-UTRs of mRNAs, respectively, is attributed to miRNAs [3, 4]. As far as their biogenesis is concerned, when miRNA sequences are transcribed, they are formulated into hairpin-like structures called pri-microRNAs [5]. The primary transcripts are initially cleaved by a RNase III enzyme known as Drosha in the nucleus, which leads to the production of precursor miRNAs (pre-miRNAs) [5]. Once the pre-miRNAs are transported into the cytoplasm, a second set of RNase III Dicer enzymes cleave the transcript to produce mature miRNAs [6]. miRNAs are associated with RNA-induced silencing complex (RISC) before they can acquire the full ability to bind their target mRNA [7]. Each miRNA can target multiple transcripts and together all the miRNAs are postulated to regulate about one-third of the human genome [8].

## 2. Deregulation of MicroRNAs in Cancer

Many human diseases, including cancer, have aberrant miRNA expression compared to normal healthy individuals [9]. In recent years, researchers have uncovered modifications at the level of genome processing. Genetic and epigenetic changes in the genome or amplification or deletion of regions can contribute to deregulation of microRNA levels [10, 11]. It has been predicted that about 45% of all pre-miRNAs have a minimum of one transcription factor binding site motif. The transcription factors can bind at conventional binding sites on the promoter of pre-miRNAs or have the ability to regulate microRNA processing by binding directly to the pri-miR and/or pre-miR [12]. An example is shown by the presence of Smad binding elements in pre-miRNAs responsive to TGF-*β*/BMP-4 stimulation [13]. Other examples for miRNA regulators include p53 [14], E2F [15], and Myc [16], the most common transcription factors for cell cycle regulation and guarding the genome. CpG hypermethylation [17] and aberrant enzymes/components of microRNA processing like Dicer or Drosha [18] also have the potential to regulate miRNAs. miRNAs can modulate all the “hallmarks of cancer” described by Hanahan and Weinberg, that is, uncontrolled proliferation, resisting apoptosis, and stimulating new pathways that can help in cancer invasion and metastasis like angiogenesis [19]. Therefore, microRNAs, although small, are critical regulators of cancer initiation and progression, and in the subsequent sections we will discuss their role in brain tumour initiation in more detail.

## 3. Cancer Stem Cell Hypothesis and Brain Tumour Initiating Cells

Many solid tumours are composed of varied cell types including vascular tissues, inflammatory cells, neighboring fibroblasts, and neoplastic/cancerous cells [20]. The majority of cells may not drive tumour formation, whereas a minority of stem cells population within the tumour is exclusively capable of self-renewal and driving tumour growth. These cells are termed as cancer stem cells (CSCs) or tumour initiating cells (TICs) [21]. This elegant concept has reemerged only recently, although its origin can be traced to 1855 [22]. The first CSCs were described in acute myeloid leukemia (AML) [23]. To prevent the tumour from relapse after standard oncology treatments, many cancer therapies are now focused on eradication of CSCs. Therefore a complete molecular and physiological understanding of CSCs in their niche is needed to achieve this goal.

After the initial discovery of prospective breast cancer stem cells [24], brain tumour initiating cells (BTICs) [25] were characterized, raising the possibility of a hierarchical organization of solid tumours. Furthermore, these rare BTICs were able to reproduce a complete heterogeneous cancer that is of similar composition as the original cancer from which it was harvested [25]. BTICs that can be distinguished, based on their abnormal stem cell-like properties, can further be identified based on markers. CD133, also referred to as prominin-1 (PROM1), is a pentaspan transmembrane cell surface protein which has gained popularity as a CSC marker [25]. The initial work applying CD133 as a BTIC marker supported CSCs to be highly associated with CD133 expression [25]. The CD133+ subpopulation within a heterogeneous GBM culture has been proven to exhibit stem-like properties such as self-renewal, high proliferation, and multipotency and at the same time this population has increased resistance to radiation treatments in comparison to CD133- GBM cells [26–29]. Although CD133 has been utilized as a BTIC marker, much is still unknown of this protein at the functional level. Further research in the past several years alluded to the discovery of additional markers such as CD15. CD15, also known as stage-specific embryonic antigen-1 (SSEA-1) or Lewis X, was initially assessed as a marker for neural stem cells (NSCs) and neural precursor cells (NPCs) in the brain [30]. In a mouse model of medulloblastoma, it was postulated that CD15+ cells were more effective at propagating tumours in comparison to CD133+ cells [31]. However, it is still conceded by multiple studies that the combination of evaluating both of these markers would provide a more accurate identification of BTICs [29, 31, 32]. The aforementioned CD133 and CD15 and even other novel proteins such as CD271 are categorized as external markers of BTICs and are used in various staining techniques to isolate them [33]. Recent research has also uncovered many cell-intrinsic markers that mark cells with tumorigenic properties. Many of these novel markers have been previously described as NSC markers. One of these, Bmi1, is a member of the polycomb group proteins, which is notably involved in self-renewal of NSCs and CSCs through means of repressing the* INK4a/ARF* locus [34]. Other more regularly used internal markers of BTICs include Sox2, FoxG1, Oct4, Twist1, and Nestin [35–38]. Nanog, a transcription factor involved in maintaining self-renewal of embryonic [39] and adult neural stem cells [40, 41], has also been shown to provide stemness in BTICs [41, 42]. Aldehyde dehydrogenase (ALDH) is an enzyme that plays a critical role in the metabolism and detoxification of external and internal substances. ALDH has also been found to be highly upregulated not only in NSCs but also in BTICs [43]. ALDH contributes to high proliferation rate and increased resistance to chemotherapy and radiation of BTICs [43]. Thus, ALDH is considered to be a BTIC marker. Other markers of BTICs include ABCG2, a key member within the ABC transporter family. This marker plays a potential role in multidrug resistance [44]. These transporters are highly expressed in CSCs and act to prevent the deterioration of these cells by means of blocking xenobiotic toxins [44].

## 4. MicroRNAs in Brain Tumour Initiating Cells

miRNAs play an important role in cellular development and growth. However, in the case of cancers, aberrant miRNA levels may play a functional role in pathogenesis. Despite evidence for the key roles that miRNAs play in brain tumour pathogenesis [45], clinically relevant knowledge of the prognostic, diagnostic, and therapeutic potential of these RNA particles in BTICs is yet to be elucidated. The implications of miRNAs in BTICs of CNS tumour for both pediatric and adult patients are discussed below.

## 5. Gliomas

Gliomas are divided according to their histological features into four grades by World Health Organization (2007) [46]. This classification from grade I to grade IV also stratifies gliomas from slow developing tumours to highly aggressive and infiltrative ones. The most aggressive grade IV glioma and accounting for almost half of the gliomas is Glioblastoma (GBM) [47]. GBM patients have a 5-year survival rate of less than 10% [48] despite advanced treatments with a combination of surgery, radiotherapy, and chemotherapy. As in the case of majority of brain tumours, selective cells within tumours are responsible for initiation and maintenance of gliomas and these are termed as glioma stem cells (GSCs) [25, 28]. The first study that described GSCs used surgical specimens of patient GBMs and characterized stem cell populations through neurosphere and clonogenic assays [49]. From then, there has been robust knowledge development in comparing normal stem cells and BTICs responsible for gliomas. These similarities are based on shared characteristics such as self-renewal, proliferation, differentiation, and expression of cell surface and internal markers including CD133, CD15, Bmi1, Nestin, Nanog, and Oct4 [29, 42, 50, 51]. Although there is some controversy with respect to the functional significance and labelling of GSCs, methods such as side population analysis [52], marker characterization, and use of* in vitro* culture [28] are useful tools for the enrichment and analysis of GSCs. Further studies of regulatory mechanisms underpinning GSCs are therefore warranted, and, more recently, there has been an increased focus on the functional role of microRNA (miRNA) in GSCs [53].

Ciafrè et al. and Chan et al. performed the first studies on miRNA expression profiles in GBM in 2005. Ciafrè et al. [54] demonstrated aberrant expression profiles of numerous miRNAs such as miR-221 which were strongly upregulated in GBM and miRNAs such as miR-128, miR-181a, miR-181b, and miR-181c which were shown to be downregulated in GBM. Chan et al. [55] also investigated the functional properties of miR-21 as an oncogenic microRNA. Since then, large-scale studies on GBM and GSCs were performed to elucidate miRNA-mediated mechanisms of tumorigenesis.

## 6. miRNAs as Tumour Suppressors in GBM

In human U251 GSCs, miR-125b was amongst the first few microRNAs found to be downregulated and shown to play a role in GSC maintenance [56, 57]. miR-125b overexpression was found to decrease the expression of cell cycle regulatory proteins, CDK6 and CDC25A, thereby preventing cell cycle arrest at the G1/S transition [56]. Another group reported the downregulation of miR-125b in CD133+ GSCs [58]. This downregulation of miR-125b leads to E2F2 expression and cell cycle progression [58]. An alternate mechanism described the effects of miR-125b on the invasion of CD133+ GSCs cells. The changes in expression of matrix metalloproteinases (MMP-2 and MMP-9) and their corresponding inhibitors (RECK and TIMP3) by miR-125b aid GSCs to infiltrate the brain [56].

Oncogenic receptor tyrosine kinases epithelial growth factor receptor (EGFR) and platelet-derived growth factor receptor-a (PDGFR) are targeted by miR-128 [59, 60]. Recent studies showed that miR-128 also modulates other mitogenic kinases such as oncogenic receptor tyrosine kinases (RTKs) in gliomas [60]. Overexpression of miR-128 is accompanied by a decrease in histone methylation H3K27me(3) and Akt phosphorylation [59]. In addition, Bmi1, a stem cell marker and an oncogene, is directly targeted by miR-128 [59]. Overexpression of miR-128 was shown to downregulate the activity of p70S6K1 and expression of its downstream signaling molecules such as HIF-1 and VEGF and reduced cell proliferation, tumour growth, and angiogenesis [61]. p70S6K1, a key downstream target of mammalian rapamycin (mTOR), is a direct target of miR-128 which plays a role in glioma tumour angiogenesis [61]. Transcription factor E2F3 was also reported as a target of miR-128 in glioma cells [62].

Many other miRNAs also function primarily as tumour suppressors. Overexpression of miR-143 inhibited glycolysis, promoted differentiation, and decreased tumorigenic capacity of GSCs* in vivo*, underscoring its important role as a tumour suppressor [63]. The expression of miR-153 that functions as an oncosuppressor was found to be downregulated in GBM tissues and in cultured CD133+ GSCs. Transfection of miR-153 into these GSCs induced differentiation and apoptosis and stalled tumour growth [64]. miR-7 was implicated in GBM as a tumor suppressor by regulating epidermal growth factor receptor (EGFR) and the Akt pathway. Moreover, miR-7 transfection was shown to decrease viability and invasiveness of GBM cells [65]. Recently, miR-145 was shown to inhibit migration and invasion of GSCs by targeting ATP-binding cassette transporter protein (ABCG2) [66]. This microRNA is normally expressed in neurons but has reduced expression in GBMs [66].

The universal anticancer gene, p53, is also linked to microRNAs in GSCs. miR-34a expression was downregulated in mutant p53 gliomas as compared with wild-type p53 gliomas [67]. miR-34a transfection inhibited survival, proliferation, cell cycle progression, cell invasion, and* in vivo* glioma xenograft growth and induces differentiation in GSCs [67, 68]. In addition, a recent report suggested that miRNA-34a regulates Akt and Wnt signaling pathways to suppress the proliferation and tumor growth of GSCs [69]. Oncogenes such as c-Met, Notch-1, and Notch-2 are the direct targets of miR-34a in GSCs [68]. Other members of miR-34 family, that is, miR-34c-3p and miR-34c-5p, also affect proliferation and invasion of glioma cells but with a differential activity [70].

Many microRNAs function effectively in modulating cell signaling. Downregulation of Notch-1 by miR-146a in GBM decreases migration of GSCs, thereby classifying miR-146a as an oncosuppressor [71]. miR-107 was reported to Notch-2 by Chen et al. in 2013 [72]. miR-326 was shown to reduce growth, invasion, and tumorigenicity of GBM stem cells by targeting Notch-1, Notch-2, and pyruvate kinase muscle enzyme (PKM2) [73]. miR-107 suppressed proliferation, downregulated stem cell markers (CD133 and Nestin), reduced MMP-12 expression, and concealed GSCs xenograft growth* in vivo* [72]. In glioma cells, miR-622 inhibited tumor invasion and migration by targeting activating transcription factor 2 (ATF2) [74].* In vivo* as well as* in vitro* data explored the role of miR-152 as a tumor suppressor in GBM stem cells [75]. Krüppel-like factor 4 (KLF4) was suggested as the downstream target of miR-152. The downregulation of KLF4 by overexpressing miR-152 leads to attenuation of the activation of MEK1/2 and PI3K signal pathways [75]. miR-218 is one of the commonly downregulated microRNAs in GSCs [76] which targets Bmi1. This again emphasizes the fact that microRNAs regulate key molecules in GSCs [76].

miR-124 and miR-137 are two oncosuppressors whose overexpression can induce differentiation and G1 cell cycle arrest of GSCs [77]. Both microRNAs bind to CDK6, a G1/S cell cycle regulator, and thereby induce differentiation of GSCs [17, 77]. Overexpression of miR-124 resulted in targeting of Neural Crest Transcription Factor Slug (SNAI2), which in turn inhibits tumorigenicity of gliomas and invasion* in vivo* [78]. Glioma pathogenesis-related protein 1(GLIPR1) or RTVP-1 is a direct target of miR-137 and this is another mechanism by which miR-137 inhibits stemness in GSCs [79]. A recent report shows the broader impact of miR-137 by regulating multiple genes in GBM. Using high throughput RNA sequencing (RNAseq) and RNA-binding protein immunoprecipitation sequencing (RIPseq), the authors have predicted miR-137 targets such as c-KIT, YBX1, Akt2, CDC42, CDK6, and TGF*β*2 [80]. miR-451 is reported to suppress tumour growth both in gliomas [81] and GBM stem cells [82].

Last but not least, members of the microRNA miR-200 family are key regulators of cell proliferation, cell cycle, and tumour growth in gliomas and GSCs. In gliomas, miR-200b suppresses tumour cell growth by targeting the CREB1 gene [83]. Low expression levels of miR-200b are linked with poor prognosis in glioma patients [84]. In gliomas, miR-200b also targets multiple members of RAB family [85]. Another member of miR-200 family, miR-200a, downregulates single-minded homolog 2-short form (SIM2-s) [86]. SIM2-s is elevated in many human cancers and in gliomas; it facilitates tumour growth and invasion [86].

## 7. miRNAs as Oncogenes in GBM

Alternative classes of microRNAs called oncomiRs or oncogenic microRNAs were found to be deregulated in GSCs. These include the miR-10 family [87] (miR-10a and miR-10b) and miR-17-92 cluster. Repressed expression of tumour suppressor genes CUB and SUSHI was observed upon upregulation of miR-10 family members [87]. As described by Guessous et al., miR-10b inhibition significantly diminishes* in vivo* tumour formation and also reduces proliferation and invasion of GSCs [88]. The first oncomiR cluster miR-17-92 [89] and miR-9 were found to be enriched in GSCs. The miR-9 target calmodulin-binding transcription activator 1 (CAMTA1) functions as a tumor suppressor [90]. The miR-17-92 cluster is associated with downregulation of CTGF and PTEN in GBM stem cells [91, 92]. OncomiR-138 has recently been identified as part of a molecular signature and prognostic biomarker of GSCs [93]. miR-330, miR-582-5p, and miR-363 promote GSC migration and invasion by reducing apoptosis [94, 95]. miR-1275 induces GSC proliferation* via* a polycomb mediated silencing mechanism and silencing of Claudin11 protein [96].

## 8. miRNAs in Pediatric Glioma

Although infrequent in children (about 15% of brain tumors), pediatric high grade gliomas (pHGG) account for high mortality [97] which depending on the key signaling pathways and copy number [98] is differentiated from adult high grade gliomas (aHGG). The deregulation of miR-129, miR-142-5p, and miR-25 in pHGG has been reported using four samples [99]. However, the high throughput microRNA study comparing pHGG, aHGG, and normal brain tissues showed the upregulation of oncogenic miR-17-92 cluster and miR-21 in pHGG [92]. Moreover, using pHGG cell lines, it was showed that the modulation of proliferation and tumorigenic signaling by miR-17-92 cluster in pHGG was by targeting tumour suppressors like PTEN and RB1.

All the above findings both in adult and pediatric gliomas suggested that microRNAs in gliomas and GSCs have a potential role in regulating fundamental core stem cell pathways and directing the fate of cancerous tissue to be either benign or malignant. A summary is provided in Table 1.

## 9. Medulloblastoma

Childhood cancers are the leading cause of disease-related mortality in children [100]. Medulloblastoma (MB) is the most common malignant brain tumour in children [100]. MB is often associated with disruption or deregulation of cerebellar development could occur in either pre- or postnatal developmental windows. MB is found to arise from cells in the external granular layer (EGL) or the subependymal matrix residing within the cerebellum [101]. MB has the potential to metastasize along the spinal cord, which portends a worse prognosis [102]. Current regimens for treatment of MB include a combination of surgery, craniospinal radiotherapy, and chemotherapy. Yet, depending on which of the 4 subgroups (Wnt, Shh, group 3, and group 4) the MB is classified into, the clinical outcome varies [102]. These four distinct groups are formulated according to the unique molecular characteristics previously described [101, 103, 104]. Despite the effectiveness of modern cancer therapies, long-term side effects are demonstrated in patients by cognition defects and developmental delays [105]. Furthermore, tumour recurrence occurs in 20 to 30% of all patients with clinical outcomes that are far worse than they were at primary diagnosis [106].

### 9.1. MicroRNAs as Tumor Suppressors in MB

miRNAs are highly associated with many cancers, and their role has also been described in MB [107]. Ferretti et al. in 2008 was one among the first to illustrate the characterization of miRNA in [108]. In their study, miR-125b, miR-324-5p, and miR-326 were demonstrated as the suppressors of sonic hedgehog signaling (Shh) pathway and MB cells showed increased proliferation when these microRNAs were diminished. The same group in 2009 depicted several microRNAs to be involved in regulation of BTICs in MBs [109]. miR-9 and miR-125 were the most promising as growth-inhibitory and apoptotic microRNAs for BTICs [109]. Another group in 2008 uncovered the role of miR-124 in MB [110]. miR-124 shows decreased expression levels in MB cells [111], with its restoration leading to inhibition of cell proliferation [110]. miR-124 is normally expressed in external granule cells of the cerebellum, which may represent a cell of origin in MB [110]. Cyclin dependent kinase 6 (CDK6) [110] and solute carrier family 16 (SLC16A1) [111] are the two identified key targets of miR-124 in MB. This suggests that miRNAs may be actively involved in the initiation and maintenance of MB through regulation of cell proliferation and cell cycle.

Role of miR-218 was also investigated in a Shh-dependent MB cell line. Cells with lower miR-218 levels presented increased tumour-like properties using both* in vitro* and* in vivo* experimental approaches [112]. Cell migration and invasion declined as miR-218 expression levels increased, suggesting miR-218 functioned as a tumour suppressor. Likewise, miR-199b-5p also functions as a tumour suppressor since overexpression of this miRNA leads to reduced tumour growth in MB [113]. Other studies further support miR-199b to be responsible for regulating BTICs within MB [114] by regulating the Notch pathway [113]. The miR-34 family was found to suppress CD15+/CD133+ BTICs. Similar to GBM, this effect was achieved by targeting the Notch-signaling pathway [115]. miR-34a overexpression in an* in vitro* study displayed inhibition of cell proliferation and cell invasion while simultaneously enriching chemotherapy sensitivity [68, 116]. Another recent study proved the role of miR-34a deficiency in accelerating MB formation* in vivo* [117].

Other studies focusing on Wnt-dependent MB show decreased expression of miR-193 and miR-224 to enhance cell proliferation and decrease radiosensitivity [118]. A small population of BTICs regulated by these microRNAs not only confers cancer stem cell characteristics in cells but also provides a means of evading therapies [25, 118, 119].

Some of the microRNAs postulated during normal embryonic and NSC development may also regulate CSCs. One such example is miR-1280. A study demonstrated the appearance of cancer stem cell-like properties in MB cells upon decreasing miR-1280 expression [120]. Downregulation of miR-1280 leads to increased Jagged-2 that causes metastatic dissemination and poor outcome in MB patients [120]. Recently, miR-135a is shown to inhibit specifically cancer stem cell driven MB by targeting Arhgef6 (rho guanine nucleotide exchange factor 6), a gene frequently upregulated in MB [121]. This emphasizes the importance of miRNA mediated targeting of BTICs.

### 9.2. OncomiRs in MB

The famous oncomiR cluster miR-17/92 family members, namely, miR-17 and miR-92, were found to be involved in Shh-dependent MB [122] as both were able to increase the proliferation rates of MB cells when overexpressed [122, 123]. This polycistronic protooncogene miR-17/92 cluster is amplified in about 6% of pediatric MB [122]. These miRNAs were shown to either induce or maintain cancer stem cells in MB. This provides evidence of aberrant hedgehog signaling contributing to the pathogenesis of MB both at the level of gene expression and the level of miRNA deregulation.

Recently miR-21, miR-96, miR-182, miR-183, and miR-142-3p [109, 124–127] were found to promote tumorigenesis and migration in BTIC of MB. The aforementioned microRNAs function by regulating proliferation, stemness, or escape from therapy. Oncosuppressor miR-128a/b [109] and oncomiRs miR-17, miR-92 [122, 123] plays a regulatory role in poor clinical outcome group 3 MB. miR-30b and miR-30d expressions may also be amplified in MB, thereby suggesting these miRNAs as putative oncogenic targets [128]. A list of microRNAs modulating BTICs in MB is compiled in Table 2. However, it is still unclear whether many of these microRNAs are capable of directly regulating only BTICs or the whole tumour cells in MB as majority of the miRNAs affect both populations.

## 10. Craniopharyngioma

Craniopharyngioma (CP) is a benign tumour of the sellar region that is commonly diagnosed in children. Due to its unpredictable growth pattern, it is often associated with severe adverse neurological effects and significant reduction in quality of life [129]. Initially it was proposed that CP can originate from either the ectopic remnants of Rathke's Pouch (RP) or embryonal epithelial cells [130, 131], but the recent development of mouse models reveals a small population of cells in CP that share the properties with stem cells [132]. Wnt/*β*-catenin pathway overactivation has been shown in these stem cells along with the high levels of secreted mitogenic signals like Shh, BMP, and FGF family. This model has provided first insight into putative CP BTICs [132]. Further studies have revealed that a fraction of cells with increased *β*-catenin levels have lower expression of Ki67 with long telomeres, both of which are the features of stem cells [133]. However, it is still unclear whether RP progenitors are truly responsible for CP initiation; therefore, a comprehensive analysis of microRNA signatures of those cells might provide the opportunity to conclusively elucidate the CP BTICs.

In 2013, Campanini et al. performed comprehensive microRNA expression profiling in CP in order to understand the role of microRNAs in CP tumorigenesis [134] (Table 3). One of the interesting findings of the paper is that, in tumours harbouring* CTNNB1* mutation, there was downregulation of miR-16 and miR-141. Furthermore,* in silico* analysis demonstrated that miR-23b, miR-24-2, miR-141, and miR-449 act as tumour suppressors by inhibiting translation of* CTNNB1* mRNA, while miR-150 was proposed to be acting as an oncomiR by modulating adenomatous polyposis coli (APC) [134]. Although there is no direct correlation between BTICs and microRNA profiling for CP in this study, it gives an outline of microRNAs that are postulated in maintenance and signaling of other types of cancer stem cells [135–137]. Further studies are required to reveal a specific miRNA expression signature that will be used to identify a more proliferative, stem-like, and likely more aggressive case of CP, thereby allowing for discovery of novel therapeutic targets to mark CP BTIC populations.

## 11. Ependymoma

Ependymoma is a common pediatric and adult CNS tumour that is thought to originate from cells lining ventricular spaces and radial glial cells of the brain [138–140]. Although chemotherapy and radiotherapy comprise the standard regimen for the treatment of these patients, the patient outcome and 5-year survival remain poor due to recurrence [139, 141]. There is still no biological marker that can be correlated with disease progression and prognosis [142]. A study by Costa et al. has identified 28 miRNAs (Table 3) that are differentially expressed in ependymoma when compared to normal ependymal cells [143]. An interesting finding emerging from the study was the identification of underexpressed miR-203 as an independent factor correlating with time to relapse, which ultimately can be used in disease management and prediction of ependymoma progression and recurrence [143]. Studies in other human cancers revealed the association of miR-203 with solid as well as hematopoietic malignancies. In leukemia, miR-203 has been implicated in the regulation of the* BCR-ABL* gene [144, 145]. Moreover, in skin, miR-203 is associated with repression of “stemness” [146]. A multivariate analysis using paraffin-embedded (FFPE) ependymoma samples from patients has identified let-7d, miR-596, and miR-367 to be associated with increased overall survival [143]. These microRNAs are shown to regulate stem cells in other cancers like breast and brain [147, 148]. Furthermore, miR-34a was shown to be overexpressed in supratentorial ependymomas which are associated with better prognosis in comparison to infratentorial tumours [143]. A signature of 5 miRNAs (miR-376a, miR-381, miR-411, miR-432, and miR-487) along with miR-203 that can be mapped to both chromosome 14q32.1 and 14q32.33 is shown in ependymoma and other tumours to be regulated by DNA methylation, proving the global dysregulation of this chromosome in carcinomas [143, 149, 150]. Furthermore, a recent work published the role of miR-376a in the regulation of human dental stem cells [151]. This further emphasized a common mode of deregulation of microRNAs in stem cells and multiple cancer types that may be affecting only a small cell population which originate these tumours.

## 12. Atypical Teratoid/Rhabdoid Tumours

Atypical teratoid/rhabdoid tumours (AT/RT) are highly aggressive tumours characterized by biallelic inactivation of the* SMARCB1/INI-1/hSNF5/BAF47* tumour suppressor gene [152, 153]. Whole exome sequencing of AT/RT tissues performed by Zhang et al. has identified a copy number decrease of the genomic locus containing* let-7a3* and* let-7b* microRNAs as well as overexpression of* HMGA2* [154]. They further showed that HMGA2 overexpression in AT/RT samples was not associated with the loss of SMARCB1. Similar to other human cancers [155, 156], the* let-7* family negatively regulates the* HMGA2* oncogene in AT/RT as well [154]. Interestingly, both overexpression of let-7a3 and let-7b and knockdown of HMGA2 resulted in decreased cell proliferation and colony formation in a rhabdoid tumour cell line [154], emphasizing the role of these microRNAs in cells selected under stem cell conditions. Although the regulation of HMGA2 by let-7 miRNAs is not unique to AT/RT and has been previously discussed in breast [157], lung [158], and ovarian [159] cancers, it provides a new therapeutic avenue in management of AT/RT tumours.

Both the aggressive nature and heterogeneity of AT/RT can suggest the existence of BTIC population responsible for tumour initiation and growth [160–162]. A fraction of cells expressing stem cell marker CD133 was shown to have an increased expression of several developmental genes including Oct 4, Sox2, Nestin, and Bmi1 [163–165]. Although several gene expression studies have been conducted in attempt to further characterize AT/RT BTICs, there is still a need for development of* in vivo* models that will help in functionally evaluating the putative BTICs and the microRNAs deregulated in them.

## 13. Pituitary Adenoma and Pilocytic Astrocytoma

Pituitary adenomas are benign tumours that account for 10–15% of all diagnosed brain malignancies [166]. The cell of origin for pituitary tumours can be one of the five differentiated cell types: growth hormone- (GH-) secreting, adrenocorticotrophic hormone- (ACTH-) secreting, prolactin- (PRL-) secreting, thyroid-stimulating hormone- (TSH-) secreting, or nonfunctioning pituitary cells (NFA) within the pituitary gland [167]. The majority of pituitary adenomas are indolent, some pituitary tumours are associated with fast growth and even metastasis to distant site such as lymph nodes and liver [166]. Although recent studies demonstrated deregulated expression of several miRNAs in the pituitary adenomas, their function and target genes remain largely unknown. A likely difference between slow growing and fast growing tumours is cell cycle regulation. Several miRNAs have been reported to regulate the cell cycle in the pituitary adenomas. As mentioned in BTICs of MB and GBM, miR-128 is also downregulated in pituitary adenomas [168]. Important cell cycle regulatory proteins such as PTEN and Bmi1 are regulated by miR-26b and miR-128, respectively [168]. miR-128a and miR-155 were found to be overexpressed in GH and NFA which resulted in the downregulation of Wee1, a known cell cycle inhibitor [169, 170]. This dual nature of miR-128 family with its different members playing both oncogenic and tumour suppressor activities can be explained based on the microRNAs processing from pri-miR to pre-miR to the export in cytoplasm [171]. Three major mechanisms suggest the differential action of miR-128 in different cell types. They are point mutation/single nucleotide polymorphism [172], loss of heterozygosity (LOH) or amplification in miR-128 host gene ARPP21 [173], and epigenetic alteration of miR-128 gene by CpG-island methylation in promoter regions [44]. Be it any mechanism, the aberrant expression of miR-128 family members needs further study to clarify its role in tumorigenesis and cancer progression [174, 175].

In addition to deregulation of cell cycle, an important hallmark of cancer is the ability of cells to evade apoptosis. miR-212 is shown to be strongly upregulated in pituitary tumours [176] and a study suggested death effector domain-containing protein (DEDD) as its potential target. This is a protein involved in apoptosis signaling [177]. Despite rare cases of metastasis of pituitary tumours, there are reports linking miRNA deregulation to tumour invasion and metastasis [178, 179]. Pituitary tumour transforming gene (PTTG) protein 1 is associated with increased tumour invasiveness [178] and is targeted by miR-126 and miR-381. These two microRNAs are downregulated in GH-secreting pituitary adenomas [179]. Furthermore, the differential expression of miRNAs is not uniform among all types of pituitary malignancies. For example, overexpression of miR-23a, miR-23b, and miR-24-2 and downregulation of miR-26b are common to GH-secreting and PRL-secreting pituitaries but are not observed in nonfunctioning adenomas [176].

Similar to pituitary adenomas, miRNA deregulation in pilocytic astrocytoma was shown to correlate with increased proliferation, migration, and evasion of apoptosis [99]. A study by Zhi et al. in 2014 demonstrated the association of miR-181b-5p downregulation with poor prognosis [180]. Increased tumor initiating cell properties were attributed to upregulated expression of neurooncological ventral antigen 1 (NOVA 1) that is normally regulated by miR-181b-5p [180], further emphasizing the role of targeting BTICs for complete eradication of cancer.

## 14. MicroRNAs: Therapeutic Applications

Treatment of cancer may eventually necessitate a multimodal approach of traditional chemotherapy/radiotherapy regimens and nontraditional RNA-based approaches. MicroRNA/RNAi based therapeutics have the potential to overcome the ineffectiveness of current treatments by either silencing the oncogenes or blocking genes that cause downregulation of oncosuppressors. This kind of novel treatment requires the discovery and complete elucidation of the functional biology of microRNAs and effective targeted delivery to their molecular targets in cancer initiating and maintaining cells. The next critical step is to gain an understanding of microRNA profiling for cancerous versus healthy cells and corresponding functional analyses. Delivery to the CNS poses another big challenge due to the difficulties in crossing the blood brain barrier and other extracellular matrix components [181]. Nevertheless, this caveat can be overcome using strategies such as nanoparticles and liposomes as encapsulated carriers [182]. Another method could be to directly target therapies in brain tumour cells using viral delivery approaches [181]. Convection-enhanced delivery can be used postsurgically to deliver RNA molecules to the tumour bed [183].

In conclusion, further developments for microRNA-mediated therapy are needed to provide clinicians with another avenue in treating the patients with deadly brain tumours. This will involve challenges in ensuring the stability and delivery of RNA therapeutics in* in vivo* systems and further depending on extent of the disease miRNAs can be used in conjunction with other available therapies.

## Figures and Tables

**Table 1:** miRNAs regulating BTICs in gliomas and GBM stem cells.

MicroRNAs	Targets	Expression in GSCs	Reference
Adult patients			
miR-128	RTKs, EGFR, PDGFR, Akt, Bmi1, HIF-1, VEGF, E2F3	Downregulated	[54, 59–62]
miR-181a, b, c	Bcl-2,	Downregulated	[54, 184]
miR-125b	CDK6, CDC25A, E2F2, MMP-2, MMP-9, RECK, TIMP3	Downregulated	[56–58]
miR-143	N-RAS, PI3K/Akt, MAPK/ERK	Downregulated	[57, 63, 185]
miR-153	Bcl-2, Mcl-1	Downregulated	[64, 186]
miR-7	EGFR, Akt	Downregulated	[65]
miR-145	ABCG2	Downregulated	[66]
miR-34a	Akt, Wnt pathway, c-Met, Notch-1, Notch-2	Downregulated	[66, 67, 69]
miR-34c-3p, 5p	Notch-2	Downregulated	[70]
miR-146a	Notch-1	Downregulated	[71]
miR-326	Notch-1, Notch-2, PKM2	Downregulated	[73]
miR-107	Notch-2, MMP-12	Downregulated	[72]
miR-622	ATF2	Downregulated	[74]
miR-152	KLF4	Downregulated	[75]
miR-218	Bmi1	Downregulated	[76]
miR-124	SNAI2, CDK6	Downregulated	[17, 77, 78]
miR-137	RTVP-1, c-KIT, YBX1, Akt2, CDC42, CDK6, TGF*β*2	Downregulated	[77, 79, 80]
miR-451	PI3K/Akt pathway	Downregulated	[81, 82]
miR-200 family	CREB1, RAB family, SIM2-s	Downregulated	[83–86]
miR-221, miR-222	p27 and p57	Upregulated	[54, 187]
miR-21	FASLG, FOXO1, IGFBP3, FBXO11	Upregulated	[55, 188–191]
miR-10 family	CUB, SUSHI	Upregulated	[87, 88]
miR-17-92 cluster	CTGF, PTEN	Upregulated	[91, 92]
miR-9	CAMTA1	Upregulated	[90]
miR-300	SH3GL2	Upregulated	[95]
miR-582-5p	Caspase3, caspase9, Bim	Upregulated	[94]
miR-363	Caspase3, caspase9, Bim	Upregulated	[94]
miR-1275	Claudin11	Upregulated	[96]

Pediatric patients			
miR-129	Undefined	Downregulated	[99]
miR-142-5p	Undefined	Upregulated	[99]
miR-25	P57	Upregulated	[99, 192]
miR-17-92	PTEN, RB1	Upregulated	[92]

**Table 2:** miRNAs regulating BTICs in MB.

MicroRNAs	Targets	Expression in MB/BTICs of MB	Reference
miR-124	CDK6	Reduced	[110, 111]
	SLC16A1		
miR-125b	Shh signaling pathway	Reduced	[108]
miR-324		Reduced	[108]
miR-326		Reduced	[108]
miR-193	Wnt signaling pathway	Reduced	[118]
miR-224		Reduced	[118]
miR-1280	JAG2	Reduced	[120]
miR-218	CDK6, RICTOR, CTSB	Reduced	[112]
miR-199b	HES1	Reduced	[113]
miR-34a	c-MET, Notch-1, Notch-2, CDK6, MAGE-A, DLL2	Reduced	[68, 116, 117]
miR-9	t-TrkC	Reduced	[109]
miR-125		Reduced	[109]
miR-128a/b	Bmi1	Reduced	[109]
miR-135a	Arhgef6	Reduced	[121]
miR-21	PDCD4	Increased	[124, 127]
miR-182/96/183	Wnt signaling pathway	Increased	[124]
miR-17/92	PTEN, Shh pathway	Increased	[109, 122, 123]
miR-142	GATA1, TAL1/E47	Increased	[125]
miR-30b/30d	Undefined	Increased	[128]

**Table 3:** miRNAs regulating BTICs in other CNS tumours.

Tumour	miRNA	Target/putative target	Expression level	References
Pituitary adenoma	miRNA-16-1	BCL1	Downregulated	[134]
	Let-7	HMGA2	Downregulated	[156]
	miR-128	Bmi1	Downregulated	[168–170]
	miR-143	ERK5	Downregulated	[169, 193]
	miR-126	PTTG1	Downregulated	[178]
	miR-381	PTTG1	Downregulated	[178]
	miR-132, miR-136, miR-127, miR-129, miR-203, miR-134, miR-127, miR-141, miR-145	N/A	Downregulated	[168, 176, 194]
	miRNA-128a	Wee1	Upregulated	[169]
	miRNA-155	Wee1	Upregulated	[169]
	miRNA-516-3p	Wee1	Upregulated	[168, 169]
	miR-26b	PTEN	Upregulated	[168–170]
	miR-26a	PKCd	Upregulated	[195]
	miR-212	DEDD	Upregulated	[176, 177]
	miR-150, miR-152, miR-191, miR-192 miR-23a, miR-23b, miR-24-2, miR-137	N/A	Upregulated	[168, 176, 194]

Craniopharyngioma	miR-141, miR-16, miR-449, miR-145, let-7a, miR-143, miR-23b, miR-15a, miR-24-2	N/A	Downregulated	[134]
	miR-150	N/A	Upregulated	[134]

AT/RT	let-7b	HMGA2	Downregulated	[99, 154]
	let-7a3	HMGA2	Downregulated	[99, 154]
	miR-140, miR-139, miR-153, miR-376b	N/A	Downregulated	[99, 154]
	miR-520b, miR-629, miR-221, miR-498, miR-373	N/A	Upregulated	[99, 154]

Pilocytic astrocytoma	miR-93, miR-135a, miR-129, miR-135b, miR-106b, miR-181b-5p	N/A	Downregulated	[99, 180]
	miR-432, miR-29a, miR-138, miR-299-5p, miR-34a	N/A	Upregulated	[99]

Ependymoma	miR-485-5p, miR-383, miR-139, miR-323, miR-433, miR-137, miR-138, miR-124a, miR-181d, miR-193b	N/A	Downregulated	[99, 143]
	miR-34a, miR-135a, miR-17-5p, miR-10a, miR-19a, miR-19b, miR-20a, miR-21, miR-32, miR-34c, miR-34b, miR-200b, miR-200a, miR-483miR-106b, miR-130a, miR-135a, miR-142-3, miR-193, miR-210, miR-301, miR-449b, miR-502, miR-518b, miR-551b, miR-565, miR-591, miR-594, miR-601	N/A	Upregulated	[99]
